# Post-Lunch Napping as a Strategy to Enhance Physiological Performance and Cognitive Function in Elite Volleyball Players

**DOI:** 10.3390/medicina60101698

**Published:** 2024-10-16

**Authors:** Özgür Eken, Oguzhan Bozkurt, Musa Türkmen, Ahmet Kurtoglu, Madawi H. Alotaibi, Safaa M. Elkholi

**Affiliations:** 1 Department of Physical Education and Sport Teaching, Faculty of Sports Sciences, Inonu University, Malatya 44000, Turkey; 2 Department of Coaching, Faculty of Sport Science, Bandirma Onyedi Eylul University, Balikesir 10200, Turkey; 3 Department of Rehabilitation Sciences, College of Health and Rehabilitation Sciences, Princess Nourah bint Abdulrahman University, P.O. Box 84428, Riyadh 11671, Saudi Arabia

**Keywords:** cognitive function, counter-movement jump, D2 attention test, physiological performance, post-lunch nap, volleyball

## Abstract

*Background and Objectives*: Volleyball demands high levels of anaerobic strength, flexibility, agility, and mental focus. Adequate sleep has been shown to enhance athletic performance and cognitive function. This study investigates the impact of post-lunch naps of varying durations (25 and 45 min) on the physiological performance and cognitive focus of elite volleyball players. *Materials and Methods*: Sixteen elite volleyball players (8 male, 8 female) with at least 7 years of competitive experience participated in a randomized crossover study. They underwent three experimental conditions: no nap (No-Nap), a 25-min nap (N25), and a 45-min nap (N45). Physical performance was measured through counter-movement jumps (CMJ) and volleyball agility tests, while cognitive function was assessed using the D2 attention test. Statistical analysis included repeated measures ANOVA to examine the differences between nap conditions and gender-specific responses. *Results*: Significant improvements in both physical performance and cognitive focus were observed in the N25 and N45 conditions compared to the No-Nap condition (indicating the effectiveness of short naps in enhancing both physical and mental performance). Males outperformed females in most physical metrics, while females scored higher on the D2 attention test (suggesting possible gender-specific responses to napping). The 25-min nap showed slightly better results in terms of sleep quality and subsequent performance (confirming that shorter naps may be more beneficial). *Conclusions*: Post-lunch naps, especially of 25 or 45 min, enhance both physiological and cognitive performance in elite volleyball players. These findings suggest that integrating short naps into athletes’ training regimens can improve performance and focus, with potential gender-specific benefits. Further research is needed to explore long-term effects and variations across other sports.

## 1. Introduction

Volleyball is a dynamic team sport that demands a high level of anaerobic strength, flexibility, speed, endurance, and agility [1]. In addition to these physical attributes, success in volleyball depends on athletes’ ability to respond quickly to the game’s unpredictable and fast-paced nature. This is especially important when executing offensive maneuvers, blocking, defense, and service reception [2]. The mastery of these sport-specific skills is critical for gaining a competitive edge and can be significantly enhanced through effective training methodologies [3]. Integrating ergogenic aids into training regimens is one such approach that has been shown to optimize athletic performance, with sleep emerging as one of the most crucial aids [4].

Sleep is important to enhance such features as physical performance. It reduces injury risk, Ref. [5] accelerates recovery, Ref. [6] and strengthens mental health, cardiovascular function, and cognitive abilities. Additionally, sleep improves immune, reproductive, and hormonal systems [7]. It is regulated by the suprachiasmatic nucleus in the hypothalamus, which controls the sleep–wake cycle. The sleep process involves adenosine, which plays multiple roles in the central nervous system, GABA receptors that reduce neural excitability, and glutamatergic receptors that activate neurons [8]. While research generally emphasizes the importance of nocturnal sleep [9], recent studies have highlighted the significant health benefits of post-lunch napping [10,11]. The optimal time for napping appears to be in the early afternoon [10,12], as this period coincides with the natural fluctuations [13] in physiological states dictated by circadian rhythms [14,15]. The increase in sleepiness during the early afternoon is often accompanied by a decline in attention, making this time ideal for rest rather than engaging in activities [16]. Whether individuals have had a normal sleep cycle or experienced sleep deprivation, taking a nap before training or competition enhances alertness [10], cognitive function [17], physical performance [12], and hormonal activity [18]. For athletes, utilizing such ergogenic aids is crucial for optimizing physical performance and achieving high levels of efficiency.

Studies have shown that taking a nap of 20, 30, or 60 min after lunch can positively impact speed, reaction time [10,12], and balance performance [11]. It also reduces sleepiness [10], regardless of whether individuals have had a normal sleep pattern or experienced sleep deprivation. For optimal performance during a 5:00 p.m. exercise session, a 25 min nap taken between 2:00 and 3:00 p.m. has been reported to be more effective in enhancing physical performance than a nap taken at 1:00 p.m. [19]. Considering factors such as prior sleep quality, nap duration, and timing, the general consensus suggests that this ergogenic aid offers substantial benefits. Given these effects, it is particularly intriguing to explore how napping influences the physical performance of volleyball players.

Post-lunch napping has been shown to increase heart rate, and electrophysiological measurements indicate that it also enhances P300 amplitude, thereby positively influencing cognitive processes [20]. Given these insights, there is a growing interest in understanding how napping, particularly after lunch, can influence the physical and cognitive performance of volleyball players. This study is crucial as it offers practical insights into the benefits of post-lunch napping. It highlights how napping can significantly enhance both physical and cognitive performance in elite athletes, particularly volleyball players, by counteracting the natural dip in performance that often occurs after lunch. The findings offer a simple, effective strategy for integrating naps into daily training regimens, potentially improving performance and reducing injury risks. Additionally, the study highlights gender-specific responses to napping, paving the way for more personalized training approaches. This study aims to examine the effects of post-lunch napping on critical performance outcomes. The hypothesis is that napping will positively impact both physical and cognitive parameters in volleyball players.

## 2. Materials and Methods

### 2.1. Participants

This study involved a carefully selected sample of 16 elite volleyball players (8 male, 8 female), each with a minimum of 7 years of competitive experience at the national level. Participants were recruited through a simple random sampling method to ensure a representative sample of experienced athletes. Inclusion criteria required that participants were actively training for at least 2 h per day, three days a week, and had maintained a consistent training regimen over the past year. All participants were between the ages of 19 and 24 years (M = 21.13, SD = 1.66) and were in peak physical condition, as indicated by their Body Mass Index (BMI) scores (M = 20.93, SD = 1.52). The demographic characteristics of the participants were recorded in pre-prepared charts, and the details are provided in Table 1.

The minimum sample size calculation in the study was performed with G-Power version 3.1.9.7 (University of Düsseldorf, Düsseldorf, Germany). Accordingly, F test (ANOVA: repeated measures, within factors) was selected. In the power analysis based on the study conducted by Kurtoğlu et al., when the effect size was taken as 0.45, α err prob = 0.05, minimum effect size = 0.33, and power (1-β err prob) = 0.80, it was determined that at least 14 participants should participate in the study [21].

#### 2.1.1. Inclusion Criteria

The inclusion criteria for the study required participants to have at least 7 years of volleyball experience, train for a minimum of 2 h per day for 3 days a week, sleep at least 8 h per night, have no history of injury or surgery in the past year, and have no habitual napping behavior [12]. 

#### 2.1.2. Exclusion Criteria

The exclusion criteria included hypertension, diabetes, cardiovascular disease, insomnia, anxiety, any acute medical condition, and the use of tobacco or alcohol. Participants who did not follow the instructions of the lead researcher or disrupted the testing process were excluded from the study. Additionally, only those students who scored ≤5 on the Pittsburgh Sleep Quality Index (PSQI) over the past month were included in the study (N = 16) [22]. 

#### 2.1.3. General Procedures

Before the commencement of the study, all participants were provided with detailed information regarding the study’s objectives, procedures, and potential risks. Informed consent was obtained from each participant in accordance with the Declaration of Helsinki. The study protocol was approved by the Non-Interventional Clinical Research Ethics Committee of İnönü University Health Sciences Institute (Approval Number: 2024/5538).

Participants were asked to maintain their usual training and dietary habits throughout the study period, with additional instructions to sleep for at least 7–8 h per night to standardize sleep conditions. Compliance with these instructions was monitored via self-reported sleep logs and verified using actigraphy data when necessary.

### 2.2. Experimental Design

This study employed a randomized, within-subject, crossover design to investigate the effects of post-lunch napping on the physiological and cognitive performance of elite volleyball players. Each participant underwent three experimental conditions: no nap (No-Nap), a 25-min nap (N25), and a 45-min nap (N45). The order of these conditions was counterbalanced across participants to control for order effects, and a one-week washout period was implemented between each condition to mitigate carryover effects. 

All sessions were conducted in a controlled indoor environment. The temperature in the gymnasium was kept constant at approximately 26 °C and maintained at an approximate humidity level of 36%. Participants were instructed to refrain from any strenuous physical activity outside of their normal training routine for 24 h prior to each session. Additionally, they were advised to maintain their usual dietary habits and consume a standardized meal at 12:00 p.m. on the day of testing, consisting of a balanced ratio of carbohydrates, proteins, and fats. Participants were advised to sleep for at least 7–8 h each night during the study period [23]. 

One week prior to the experimental sessions, participants attended a familiarization session to become acquainted with the testing procedures, equipment, and environment. During this session, participants were instructed on the proper execution of the counter-movement jump (CMJ) and the D2 attention test, measurement scales, and equipment [12]. 

**Figure 1:**
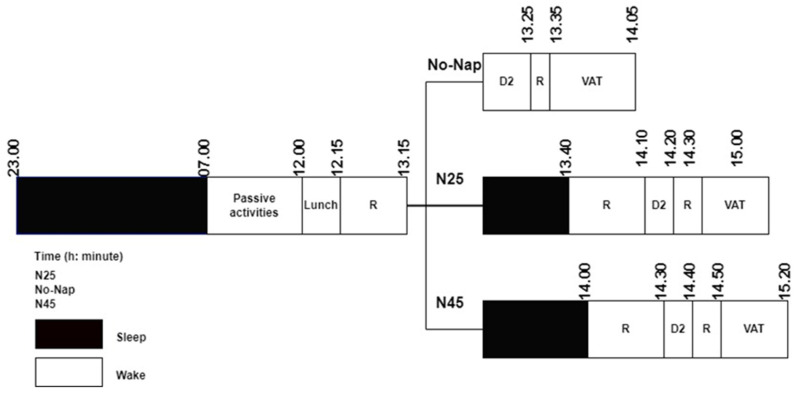
Experimental design of study: (No-nap: no post-lunch napping, N25: 25 min post-lunch napping, N45: 45 min post-lunch napping, R: rest, D2: D2 attention test, VAT: volleyball agility test).

The session began with the expert trainer demonstrating and guiding the participants through the warm-up protocol and the volleyball agility test. Participants then repeated the procedures to ensure they could perform the test accurately. The warm-up protocol included 3 min of jogging at approximately 40% of the target heart rate, followed by dynamic stretching, jumping, and high-intensity sprints, lasting a total of 10 min [24]. The target heart rate was calculated using the Karvonen formula ((220 − Age − Resting Heart Rate) × Exercise Intensity + Resting Heart Rate) [25]. During the warm-up, participants’ heart rates were monitored using the Polar RS400 device. After ensuring a normal night’s sleep (at least 8 h), participants completed three test protocols—No-Nap, 25-min nap (N25), and 45-min nap (N45) each separated by a week. The experimental protocol for the study is outlined in Figure 1.

#### 2.2.1. No-Nap Protocol

On the day of testing, participants were awakened at 7:00 a.m. Between 7:00 a.m. and 12:00 p.m., they engaged in leisure activities that did not involve strenuous physical exertion, such as reading or walking. At 12:00 p.m., they had a standard lunch, followed by a 60 min rest period during which they were restricted from using electronic devices, including phones, televisions, computers, and tablets. At 1:15 p.m., participants took a cognitive attention test, followed by a 10 min break. From 1:35 p.m.to 2:05 p.m., they completed the volleyball agility test. On the day of testing, participants were allowed to consume only water, except for the standard lunch.

#### 2.2.2. Nap-25 Protocol

On the day of testing, participants were awakened at 7:00 a.m. From 7:00 a.m. to 12:00 p.m., they engaged in leisure activities that did not involve strenuous physical activity, such as reading or walking. At 12:00 p.m., they consumed a standard lunch, followed by a 60 min rest period during which their exposure to electronic devices, including phones, televisions, computers, and tablets, was restricted. At 1:05 p.m., participants entered a pre-prepared sleep room that was comfortable, warm (26 ± 1.6 °C), dark, and quiet (humidity 36 ± 3.4%). They were given 10 min to acclimate to the environment (Romdhani et al., 2021 [12]). After this acclimation period, participants wore eye masks and earplugs and took a 25 min nap from 1:15 p.m. to 1:40 p.m. They rated their sleep experience on a scale from 0 to 10, where 0 indicated “no sleep”, 5 indicated “partial sleep”, and 10 indicated “high-quality, deep sleep” [26]. To overcome sleep inertia, participants were given a 30 min recovery period from 1:40 p.m. to 2:10 p.m. [10]. Following this, a cognitive attention test was administered from 2:10 p.m. to 2:20 p.m. After a 10 min break, they proceeded to the volleyball agility test, which was conducted from 2:30 p.m. to 3:00 p.m. On the testing day, participants were allowed to consume only water, aside from their lunch.

#### 2.2.3. Nap-45 Protocol

On the day of testing, participants were awakened at 7:00 a.m. From 7:00 a.m. to 12:00 p.m., they engaged in leisure activities that did not involve strenuous physical activity, such as reading or walking. At 12:00 p.m., they had a standard lunch, followed by a 60 min rest period during which they were restricted from using electronic devices such as phones, televisions, computers, and tablets. At 1:05 p.m., participants entered a pre-prepared sleep room that was comfortable, warm (25 ± 1.4 °C), dark, and quiet (humidity 33 ± 2.8%). They were given 10 min to acclimate to the environment [12]. After this acclimation period, participants wore eye masks and earplugs and took a 45 min nap from 1:15 p.m. to 2:00 p.m. They rated their sleep experience on a scale from 0 to 10, where 0 indicated “no sleep”, 5 indicated “partial sleep”, and 10 indicated “high-quality, deep sleep” [26]. To overcome sleep inertia, participants were given a 30 min recovery period from 2:00 p.m. to 2:30 p.m. [10]. Following this, a cognitive attention test was administered from 2:30 p.m. to 2:40 p.m. After a 10 min break, participants proceeded to the volleyball agility test, which was conducted from 2:50 p.m. to 3:20 p.m. On the testing day, participants were allowed to consume only water, aside from their lunch.

### 2.3. Data Collection Tools

Participants’ height was measured using a Seca model 213 (Hamburg, Germany) with an accuracy of 5 mm, and their weight was measured using a Seca model 813 (Birmingham, UK) with an accuracy of 0.1 kg. These measurements were recorded in pre-prepared charts. Leg length was measured with a tape measure in two positions: (1) with the knee joint in extension and the ankle joint in plantar flexion, from the greater trochanter of the femur to the distal end of the first phalanx of the foot, and (2) in a squatting position with the hip and knee joints at 90 degrees, from the greater trochanter of the femur to the posterior aspect of the calcaneus and the point where it contacts the ground. These measurements were recorded accordingly.

#### 2.3.1. Pittsburg Sleep Scale

The Pittsburgh Sleep Quality Index (PSQI) was used to determine whether the participants included in the study had any sleep disorders. A score greater than 5 on the scale indicates the presence of a sleep disorder [22]. 

#### 2.3.2. Subjective Sleepiness Scale

Participants rated their sleep experience using a subjective scale ranging from 0 to 10, where a score of 0 indicated “no sleep”, 5 indicated “partial sleep”, and 10 indicated “high-quality, deep sleep” [26].

#### 2.3.3. D2 Attention Test

The D2 attention test was used to measure participants’ attention skills. This test is advantageous because it can be administered to multiple individuals simultaneously and completed in a short amount of time. The task involves identifying the letter “d” with two dots among 14 rows of “d” and “p” letters. Participants were given 20 s per row to complete the task, with the entire test taking 8 min. Including the preparation phase, the test took a total of 10 min in our study [27].

#### 2.3.4. Volleyball Agility Test

The test was conducted in the area between the 3 m line and the net. Upon the signal from the lead researcher, participants performed a spike jump and then moved forward to start the stopwatch. They then shifted to the right, executing block jumps on balls positioned 1 m to the left and 1 m to the right of the court’s center. After completing these jumps, participants continued to the right side of the court, turned, and performed two more block jumps. Finally, they sprinted diagonally, passing by the photocell located on the 3 m line and 3 m from the court’s sideline, which stopped the stopwatch. Participants’ jumping performance was recorded using a Canon camera placed on the right sideline of the court. Additionally, spike jumps were measured over a net height of 239 cm. The time between two photocells was recorded in seconds, and jump heights were measured in centimeters [28]. The collected data were recorded and analyzed using the My Jump Lab iOS application [29]. 

### 2.4. Statistical Analysis

In this study, SPSS software (IBM, version 25, Chicago, IL, USA) was used for statistical analyses. Normality analysis of the data was performed using the Shapiro–Wilk test and it was confirmed that the data followed a normal distribution. Levene’s test was performed to evaluate the homogeneity of variances. The repeated measures ANOVA test was performed to analyze the interaction of post-lunch napping with physiological and cognitive functions between genders. In addition, the TUKEY post hoc test was applied to determine the differences between the tests. The results of the ANOVA test were determined according to Mauchly’s Test of Sphericity. If Mauchly’s Test of Sphericity yielded a value greater than 0.05, the assumptions of sphericity were considered to be met; otherwise, the Greenhouse–Geisser correction was applied. Effect sizes were calculated using Cohen’s d formula to determine the magnitude of findings. For ANOVA, effect size was determined based on partial eta squared (ηp2) values; ηp2 values indicate effect size as follows: ηp2 ≤ 0.01 indicates small effect size, 0.01 ≤ ηp2 ≤ 0.06 indicates medium effect size, and ηp2 ≥ 0.14 indicates large effect size [30]. GraphPad Prism version 8 (GraphPad Software, Inc., San Diego, CA, USA) was used to visualize the data. The significance level was set as 0.05 for this study. Since the data were normally distributed, the evaluations were presented as mean (M) and standard deviation (SD).

## 3. Results

The demographic characteristics of the study participants, including age, weight, height, body mass index (BMI), and leg length, are presented separately for male and female volleyball players (Table 2). The average age of male participants was 22.1 ± 1.5 years, and for females, it was 20.1 ± 1.1 years. Males had an average weight of 71.8 ± 6.7 kg and a height of 183.0 ± 3.5 cm, while females had an average weight of 58.7 ± 6.3 kg and a height of 169.5 ± 6.0 cm. The BMI for males averaged 21.4 ± 1.6 kg/m² and for females 20.4 ± 1.3 kg/m². The average leg length was 113.0 ± 3.5 cm for males and 104.0 ± 5.6 cm for females, while leg length measured at 90° flexion averaged 79.2 ± 2.7 cm for males and 76.3 ± 5.3 cm for females. These measurements provide a comprehensive overview of the physical attributes of the study’s male and female participants, offering insights into their potential impact on performance-related outcomes.

**Figure 2:**
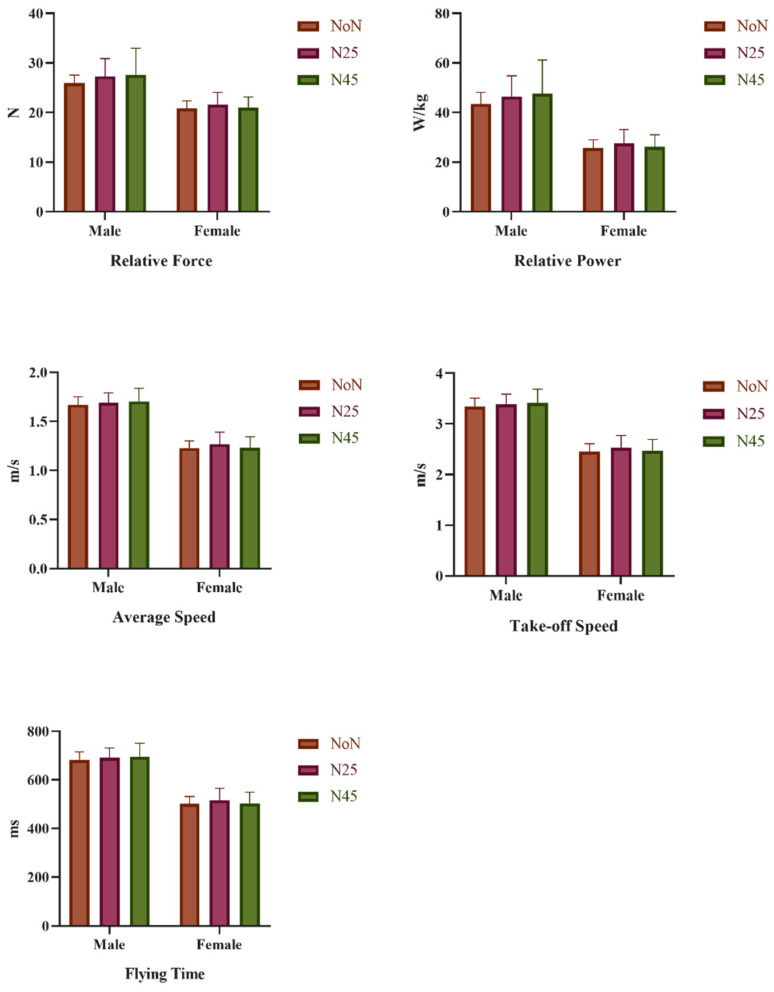
Investigation of CMJ performance parameters of participants according to napping time.

In Table 3, the anaerobic performance parameters (CMJ, force, power, RP, AS, TS, impulse, and FT), D2 test results, and repeated measures ANOVA test results of the participants after NoN, N25, and N45 were analyzed. Although no significant difference was detected in vertical jump performance parameters, there was a significant difference between N25 and N45 in RF results (mean difference = 0.529, std. err. = 0.210, *p* = 0.030 (95% CI = 0.06 to 0.99)), between N25 and N45 in RP results (mean difference = 1.35, std. err. = 0.576, *p* = 0.041 (95% CI = 0.06 to 2. 63)), between N25 and N45 in AS results (mean difference = 0.029, std. err. = 0.011, *p* = 0.028 (95% CI = 0.004 to 0.055)), between N25 and N45 in TS results (mean difference = 0.055, std. err. = 0.022, *p* = 0.032 (95% CI = 0.006 to 0.104)), and FT outcomes (mean difference = 0.055, std. err. = 0.022, *p* = 0.032 (95% CI = 0.006 to 0.104)) (Figure 2).

**Figure 3:**
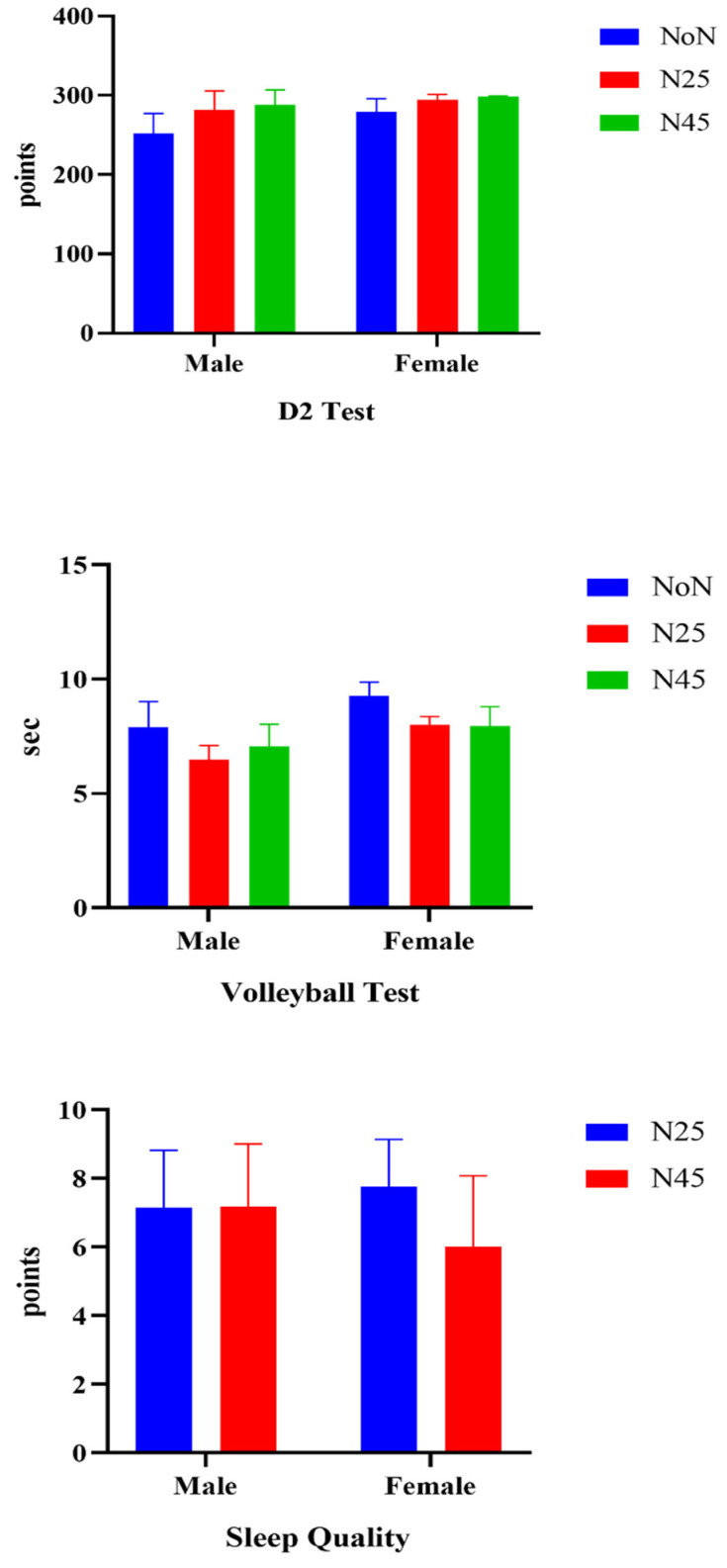
Investigation of D2, volleyball test and sleep quality of participants.

In addition, males obtained higher results than females in all parameters except D2 test results (*p* < 0.05). There was a statistically significant difference between the participants’ D2 test results (F(1, 9) = 12.883, ηp2 = 617, *p* = 0.005) and volleyball test results (F(1, 9) = 19.111, ηp2 = 680, *p* < 0.001) in the time interaction. In the post hoc test (TUKEY), there was a significant difference between NoN and N25 (mean difference = −19.9, std. err. = 5.1, *p* = 0.005), and between NoN and N45 (mean difference = 23.0, std. err. = 6.3, *p* = 0.007) in the D2 test results. Also, according to the volleyball test results, there was a significant difference between NoN and N25 (mean difference = 1.4, std. err. = 0.197, *p* = 0.001), and between NoN and N45 (mean difference = 0.913 std err. = 0.15, *p* = 0.007). When the sleep scores of the participants were analyzed, while there was no difference in the time interaction (*p* > 0.05), in the group × time interaction, sleep quality increased at N25, while sleep quality decreased after N45 (F(1, 9) = 7512, ηp2 = 429, *p* = 0.021) (Figure 3). There was also no significant difference in the group × time interaction (*p* > 0.05).

## 4. Discussion

The results of this study demonstrate that post-lunch napping has a significant impact on both the physiological performance and cognitive function of volleyball players. The findings are consistent with previous research, indicating that napping can serve as an effective ergogenic aid, particularly in enhancing athletic performance and attention during subsequent physical activities [10,12,19,26,31,32,33,34,35,36,37,38]. 

Observed improvements in counter-movement jump performance, force, and power metrics in the N25 and N45 protocols were indicative that brief afternoon naps may optimally enhance muscle recovery. These naps also increased the explosive power of athletes. This is particularly relevant for volleyball players, where quick, explosive movements are critical for performance [39,40]. The significant differences in the volleyball agility test results highlight the benefits of napping. It improves neuromuscular coordination and agility, which are essential components of volleyball performance. There is only one study that examine the effect of napping on agility performance [41]. Souabni et al. [41] and our study both highlight the significant impact of napping on enhancing physiological performance in athletes, yet they differ in scope, methodology, and specific findings. Souabni et al. demonstrated that a 40 min nap improved defensive and offensive agility, upper body power, and reduced stress and fatigue in elite basketball players, leading to enhanced performance across multiple metrics [42]. Similarly, our study found that post-lunch naps of 25 and 45 min significantly enhanced both physical performance, as measured by counter-movement jump (CMJ) metrics, and cognitive function, assessed through the D2 attention test, in elite volleyball players. Notably, our findings also revealed gender differences, with male athletes outperforming females in most physical performance metrics, except for the D2 test, suggesting potential gender-specific responses to napping that merit further investigation. The contrast in nap duration and the sport-specific context between the two studies—basketball versus volleyball—underscores the importance of tailored napping protocols.

Cognitive function, measured by the D2 attention test, showed significant improvement after both the 25 and 45 min naps. This supports the hypothesis that post-lunch naps can reduce the cognitive decline typically experienced during the post-lunch dip. As a result, athletes are better able to maintain focus and reaction time during demanding tasks. There are many other studies that examine the effect of napping on cognitive function [19]. Abdessalem et al. [19] found that while 25 min naps taken at 14:00 h and 15:00 h improved physical performance, there were no significant differences in vigilance, as measured by the digit cancellation test, across different nap timings or in comparison to the no-nap condition. This suggests that the cognitive benefits of napping may be less influenced by the timing of the nap and more dependent on other factors [19,42,43]. In contrast, our study focused on post-lunch naps of varying durations (25 and 45 min) and their effects on cognitive function in elite volleyball players, as assessed by the D2 attention test. We observed significant improvements in cognitive focus in both nap conditions compared to the no-nap condition. This indicates that nap duration plays a crucial role in enhancing cognitive performance. Notably, this improvement was observed regardless of the time of day, suggesting that nap duration may be a more important factor than timing when it comes to cognitive benefits. Boukhris et al. [44] demonstrated that both 40 min and 90 min naps significantly improved attention in trained male athletes, with the longer 90 min nap yielding superior gains in cognitive performance [44]. This suggests that extended nap durations may offer enhanced cognitive benefits, potentially due to the opportunity to enter deeper stages of sleep, which are known to support cognitive recovery and function. In contrast, our study examined the effects of shorter, post-lunch naps—25 and 45 min—on the cognitive performance of elite volleyball players, as measured by the D2 attention test. We found that even these shorter naps significantly enhanced cognitive focus compared to the no-nap condition, indicating that substantial cognitive benefits can be achieved within a more constrained time frame. The cognitive results from both studies emphasize the effectiveness of napping in enhancing attention and mental focus. They also highlight the flexibility of nap duration in achieving these benefits. While Boukhris et al. [44] emphasize the greater cognitive improvements associated with longer naps, our findings suggest that shorter naps can still be highly effective, offering a more practical option for athletes needing quick cognitive recovery. 

Fang et al. [43] conducted a comprehensive analysis of 4535 studies, focusing on 20 reports that specifically examined the relationship between napping and cognitive impairment. Their pooled analysis revealed a significant association between napping and an increased risk of dementia. Notably, the risk of cognitive impairment was found to be substantially higher among individuals who napped for extended durations. Those who napped for more than 30, 45, and 60 min per day exhibited a 35%, 41%, and 40% greater likelihood, respectively, of developing cognitive impairment [43]. These findings underscore the potential cognitive risks associated with prolonged napping.

Interestingly, while both male and female participants benefited from napping, the data showed that male participants generally outperformed females in most physiological parameters. However, in the D2 test, females had slightly higher scores. This may suggest a gender-specific response to napping, potentially influenced by differences in muscle mass, metabolism, or other physiological factors [45,46]. Hormones such as estrogen may affect women’s cognitive functions, leading to better performance in cognitive domains such as attention and memory [47]. It has also been suggested that women generally exhibit greater resilience to stress and anxiety, which may contribute to their better performance in tasks requiring attention [48]. Downing et al. [49] reported that women generally have higher levels of attention and self-regulation abilities [49]. In addition, women are known to outperform men in language and verbal skills, while men are better at visual-spatial tasks, which has been supported by research [50]. Additionally, women are reported to have greater gray matter density in brain areas associated with attention and memory. This may enhance their performance in cognitive tasks [51]. It has also been suggested that women tend to use better strategies in cognitive tasks, which may contribute to their higher scores on the D2 test [52].

Moreover, the comparison between the N25 and N45 protocols showed that both nap durations were effective. However, the 25 min nap was slightly more beneficial in terms of sleep quality and subsequent performance. This aligns with previous studies suggesting that shorter naps can prevent sleep inertia and are more effective in enhancing performance during the late afternoon [21,42,53]. Kurtoğlu et al. [21] examined the effects of nap duration on respiratory function in young elite athletes, finding that a 45 min nap (N45) significantly enhanced peak expiratory flow (PEF) [21]. Similarly, our study demonstrated that both 25 and 45 min post-lunch naps (N25 and N45) led to significant improvements in both physical performance and cognitive focus in elite volleyball players. Both studies underscore the potential of strategic napping to optimize various aspects of athletic performance. However, while Kurtoğlu et al. [21] focused on respiratory function, our research assessed both physiological and cognitive outcomes, offering a broader perspective on the diverse benefits of napping across different sports performance indicators. These findings collectively support the positive impact of napping on different physiological systems, while also suggesting that the effects of napping may vary depending on the sport and the specific performance parameters being evaluated. 

Boukhris et al. [44] investigated the impact of daytime napping following normal nighttime sleep on physical performance in physically active individuals and athletes, highlighting that napping generally improved performance metrics such as the highest distance and total distance in the 5 m shuttle run test (5MSRT), while reducing fatigue [42]. However, they found no significant effects on muscle force, sprint performance, or the 30 s Wingate test in most studies reviewed. In contrast, our study focused on the effects of post-lunch napping on both physiological and cognitive performance in elite volleyball players, demonstrating significant improvements in counter-movement jump (CMJ) metrics and cognitive focus, particularly following 25 and 45 min naps. While Boukhris et al.’s [44] review emphasizes the physical benefits of napping in various activities, our findings extend the discussion by underscoring the dual benefits of napping on both physical and cognitive performance in a specific athletic context. Adanur and Eken [53] examined the effects of short-duration napping on anaerobic performance in young elite kickboxers, focusing on metrics such as mean and peak power outputs [53]. Their findings revealed that while counter-movement jump (CMJ) values were largely unaffected by napping, significant improvements in power outputs were observed, particularly after 25 and 45 min naps. Similarly, our study examined the effects of post-lunch napping on both physiological performance and cognitive function in elite volleyball players. Significant improvements were observed in both physical performance and cognitive focus following naps of similar durations. Both studies highlight the potential of napping as an effective strategy to enhance athletic performance, especially in sports that require high anaerobic power.

Ajjimaporn et al. [31] investigated the effects of a 20 min nap after sleep deprivation on various performance and brain activity metrics in college soccer players, finding that while the nap partially restored leg muscle strength and anaerobic sprint power, it did not fully reverse the cognitive and physical deficits caused by sleep deprivation [34]. Similarly, our study examined the impact of post-lunch napping on physiological and cognitive performance in elite volleyball players, showing significant improvements in both physical and cognitive metrics following 25 and 45 min naps. While Ajjimaporn et al.’s study highlights the limitations of short naps in fully mitigating the effects of sleep deprivation, our research demonstrates the efficacy of slightly longer naps in enhancing overall performance. Both studies underscore the importance of napping in athletic performance but also suggest that the duration and context of the nap play crucial roles in determining its effectiveness.

Despite the valuable findings of this study, there are a few limitations that should be acknowledged. First, the sample size was relatively small (16 participants), limiting the generalizability of the results. Larger studies involving a broader range of athletes from different sports and age groups are needed to validate these findings. Second, the study focused only on the short-term effects of post-lunch napping. Longitudinal studies examining the long-term benefits and potential detriments of regular napping on performance are required to draw more comprehensive conclusions. Additionally, the specific nap durations (25 and 45 min) may not capture the full range of possible benefits or effects, and future research should explore different nap lengths to optimize performance outcomes. Lastly, the study did not account for individual differences in chronotype, which may influence the effectiveness of naps based on the time of day. 

The results of this study provide practical insights for coaches, athletes, and sports performance practitioners. Incorporating short post-lunch naps (25–45 min) into training regimens could serve as an effective strategy to enhance both physiological and cognitive performance, especially in sports requiring high anaerobic power, agility, and mental focus, such as volleyball. The findings suggest that post-lunch napping could be used as a simple and accessible tool to mitigate the typical afternoon performance decline, promoting recovery and optimizing readiness for subsequent training or competition. Coaches may also consider gender-specific responses when designing nap protocols, as males and females demonstrated differences in performance outcomes.

Further research is essential to expand on the findings of this study. Future studies should investigate the effects of post-lunch naps on athletes from various sports and at different competitive levels to assess whether the observed benefits are sport-specific or generalizable across athletic populations. Additionally, exploring the influence of individual chronotypes and different nap timings on both physical and cognitive performance would provide deeper insights into optimizing nap strategies for each athlete. Research on the long-term effects of regular post-lunch napping on performance, recovery, and injury prevention could also yield significant contributions to sports science and athlete management. Finally, combining napping with other recovery modalities, such as nutritional interventions and active recovery, could further enhance athletes’ overall performance and well-being.

## 5. Conclusions

This study demonstrates that post-lunch naps of 25 to 45 min significantly enhance both physiological performance and cognitive function in elite volleyball players. Integrating short naps into training regimens offers a practical strategy to boost athletic performance and mental focus.

## Figures and Tables

**Table 1:** Demographic characteristics of the participants.

Variables	N	Minimum	Maximum	M + S.D.
Age (year)	16	19	24	21.13 ± 1.66
Height (cm)	16	160	187	176.25 ± 8.43
Weight (kg)	16	48	82	65.31 ± 9.30
BMI (kg/m^2^)	16	18.8	24.2	20.93 ± 1.52

BMI: Body Mass Index.

**Table 2:** Demographic information of participants.

Parameters	Male	Female
Age (years)	22.1 ± 1.5	20.1 ± 1.1
Weight (kg)	71.8 ± 6.7	58.7 ± 6.3
Height (cm)	183.0 ± 3.5	169.5 ± 6.0
BMI (kg/m^2^)	21.4 ± 1.6	20.4 ± 1.3
Leg length (cm)	113.0 ± 3.5	104.0 ± 5.6
Leg length at 90° flexion (cm)	79.2 ± 2.7	76.3 ± 5.3

BMI: Body Mass Index.

**Table 3:** Investigation of participants’ performance test results according to napping time.

Parameters	Time	Male	Female	Group	Time	Group × Time
		M ± SD	M ± SD	MD	F	F
				Std. Err.	ηp^2^	ηp^2^
				*p*	*p*	*p*
CMJ (cm)	NoN	56.6 ± 6.5	31.0 ± 4.0	24.28	1.281	1.117
	N25	56.4 ± 7.0	33.8 ± 6.0	3.2	0.114	0.105
	N45	55.98 ± 6.45	31.3 ± 5.5	<0.001	0.3	0.329
Force (N)	NoN	1882.9 ± 189.7	1240.1 ± 186.6	614.6	1.193	1.372
	N25	1874.1 ± 135.8	1299.5 ± 201.5	103.2	0.107	0.121
	N45	1868.7 ± 181.9	1242.3 ± 174.6	<0.001	0.324	0.276
RF (N)	NoN	25.8 ± 1.8	20.9 ± 1.6	4.47	1.677	1.687
	N25	25.8 ± 1.8	21.9 ± 2.3	1.09	0.144	0.144
	N45	25.7 ± 1.7	21.0 ± 2.1	0.002	0.212 ^c^	0.21
Power (W)	NoN	3133.2 ± 366.4	1533.2 ± 294.7	1571.7	0.011	0.035
	N25	3107.7 ± 221.8	1536.7 ± 677.1	204.9	0.001	0.003
	N45	3089.8 ± 336.3	1545.4 ± 294.7	<0.001	0.989	0.966
RP (W/kg)	NoN	43.2 ± 5.5	25.8 ± 3.5	16.1	1.429	1.309
	N25	43.0 ± 5.6	28.4 ± 5.3	2.7	0.125	0.116
	N45	42.6 ± 5.4	26.1 ± 4.8	<0.001	0.263 ^c^	0.292
AS (m/s)	NoN	1.6 ± 0.09	1.2 ± 0.07	0.409	1.662	1.568
	N25	1.6 ± 0.1	1.2 ± 0.1	0.05	0.1432	0.136
	N45	1.6 ± 0.09	1.2 ± 0.1	<0.001	0.215 ^c^	0.233
TS (m/s)	NoN	3.3 ± 0.1	2.4 ± 0.1	0.82	1.516	1.412
	N25	3.3 ± 0.2	2.5 ± 0.2	0.114	0.132	0.124
	N45	3.3 ± 0.1	2.4 ± 0.2	<0.001	0.244 ^c^	0.267
Impulse (kg × m/s)	NoN	242.1 ± 20.9	145.7 ± 20.0	93.6	1.132	1.198
	N25	241.3 ± 15.8	151.6 ± 20.8	11.1	0.102	0.107
	N45	240.7 ± 20.0	145.6 ± 18.6	<0.001	0.342	0.323
FT (ms)	NoN	678.4 ± 40.2	501.8 ± 32.6	167.2	1.517	1.362
	N25	677.4 ± 42.4	523.5 ± 48.5	23.1	0.132	0.12
	N45	674.8 ± 38.9	503.5 ± 45.9	<0.001	0.243^c^	0.279
D2 test (points)	NoN	269.6 ± 2.0	278.5 ± 1.7	−3.3	12.883	0.497
	N25	294.3 ± 1.5	293.8 ± 7.6	0.5.3	0.617	0.058
	N45	296.3 ± 1.5	298.0 ± 1.1	0.546	0.005 ^a,b^	0.524
Volleyball test (s)	NoN	7.0 ± 0.3	9.3 ± 0.6	−1.77	19.111	3.314
	N25	6.2 ± 0.5	8.0 ± 0.3	0.0.3	0.68	0.269
	N45	6.6 ± 0.5	7.9 ± 0.8	<0.001	<0.001 ^a,b^	0.06
Sleep score (points)	N25	6.8 ± 1.7	7.7 ± 1.4	0.043	4.7	7.512
				1.024	0.32	0.429
	N45	7.0 ± 2.0	6.0 ± 2.0	0.967	0.055	0.021

CMJ: counter-movement jump, RF: relative force, RP: relative power, AS: average speed, TS: take-off speed, FT: flying time, ^a^ = difference between NoN–N25, ^b^ = difference between NoN–N40, ^c^ = difference between N25–N40.

## Data Availability

The datasets generated and/or analyzed during the current research are available from the corresponding author on reasonable request.

## References

[1] Bonato M., De Capitani M.C., Banfi G. (2022). Agility Training in Volleyball. J. Sports. Med. Phys. Fit..

[2] Frohner B., Cengiz A. (1999). Volleyball Game Theory and Exercises.

[3] Silva A.F., Clemente F.M., Lima R., Nikolaidis P.T., Rosemann T., Knechtle B. (2019). The Effect of Plyometric Training in Volleyball Players: A Systematic Review. Int. J. Environ. Res. Public Health.

[4] Khemila S., Abedelmalek S., Romdhani M., Souissi A., Chtourou H., Souissi N. (2021). Listening to Motivational Music during Warming-up Attenuates the Negative Effects of Partial Sleep Deprivation on Cognitive and Short-Term Maximal Performance: Effect of Time of Day. Chronobiol. Int..

[5] Charest J., Grandner M.A. (2020). Sleep and Athletic Performance. Sleep Med. Clin..

[6] Nédélec M., Halson S., Abaidia A.-E., Ahmaidi S., Dupont G. (2015). Stress, Sleep and Recovery in Elite Soccer: A Critical Review of the Literature. Sports Med..

[7] Baranwal N., Yu P.K., Siegel N.S. (2023). Sleep Physiology, Pathophysiology, and Sleep Hygiene. Prog. Cardiovasc. Dis..

[8] Luppi P.-H., Fort P. (2019). Sleep–Wake Physiology. Handbook of Clinical Neurology.

[9] Reutrakul S., Van Cauter E. (2018). Sleep Influences on Obesity, Insulin Resistance, and Risk of Type 2 Diabetes. Metabolism.

[10] Waterhouse J., Atkinson G., Edwards B., Reilly T. (2007). The Role of a Short Post-Lunch Nap in Improving Cognitive, Motor, and Sprint Performance in Participants with Partial Sleep Deprivation. J. Sports Sci..

[11] Ammar A., Boukhris O., Hsouna H., Ben Dhia I., Trabelsi K., Ali Gujar T., Clark C.C.T., Chtourou H., Driss T., Hoekelmann A. (2021). The Effect of a Daytime 60-Min Nap Opportunity on Postural Control in Highly Active Individuals. Biol. Sport.

[12] Romdhani M., Dergaa I., Moussa-Chamari I., Souissi N., Chaabouni Y., Mahdouani K., Abene O., Driss T., Chamari K., Hammouda O. (2021). The Effect of Post-Lunch Napping on Mood, Reaction Time, and Antioxidant Defense during Repeated Sprint Exercice. Biol. Sport.

[13] Monk T.H. (2005). The Post-Lunch Dip in Performance. Clin. Sports Med..

[14] Scheer F.A.J.L., Hu K., Evoniuk H., Kelly E.E., Malhotra A., Hilton M.F., Shea S.A. (2010). Impact of the Human Circadian System, Exercise, and Their Interaction on Cardiovascular Function. Proc. Natl. Acad. Sci. USA.

[15] Wolff C.A., Esser K.A. (2019). Exercise Timing and Circadian Rhythms. Curr. Opin. Physiol..

[16] Bes F., Jobert M., Schulz H. (2009). Modeling Napping, Post-Lunch Dip, and Other Variations in Human Sleep Propensity. Sleep.

[17] Slama H., Deliens G., Schmitz R., Peigneux P., Leproult R. (2015). Afternoon Nap and Bright Light Exposure Improve Cognitive Flexibility Post Lunch. PLoS ONE.

[18] Vgontzas A.N., Pejovic S., Zoumakis E., Lin H.M., Bixler E.O., Basta M., Fang J., Sarrigiannidis A., Chrousos G.P. (2007). Daytime Napping after a Night of Sleep Loss Decreases Sleepiness, Improves Performance, and Causes Beneficial Changes in Cortisol and Interleukin-6 Secretion. Am. J. Physiol. Endocrinol. Metab..

[19] Abdessalem R., Boukhris O., Hsouna H., Trabelsi K., Ammar A., Taheri M., Irandoust K., Hill D.W., Chtourou H. (2019). Effect of Napping Opportunity at Different Times of Day on Vigilance and Shuttle Run Performance. Chronobiol. Int..

[20] Petit E., Bourdin H., Tio G., Yenil O., Haffen E., Mougin F. (2018). Effects of a 20-Min Nap Post Normal and Jet Lag Conditions on P300 Components in Athletes. Int. J. Sports Med..

[21] Kurtoğlu A., Eken Ö., Aydın E., Çar B., Nobari H. (2024). The Impact of Strategic Napping on Peak Expiratory Flow and Respiratory Function in Young Elite Athletes. BMC Sports Sci. Med. Rehabil..

[22] Buysse D.J., Reynolds C.F., Monk T.H., Berman S.R., Kupfer D.J. (1989). The Pittsburgh Sleep Quality Index: A New Instrument for Psychiatric Practice and Research. Psychiatry Res..

[23] Reilly T., Atkinson G., Edwards B., Waterhouse J., Farrelly K., Fairhurst E. (2007). Diurnal Variation in Temperature, Mental and Physical Performance, and Tasks Specifically Related to Football (Soccer). Chronobiol. Int..

[24] Ribeiro Y.S., Del Vecchio F.B. (2011). Meta-analysis of the acute effects of stretching on high intensity sprint performance. Rev. Bras. Educ. Fis. Esporte..

[25] Karvonen M.J., Kentala E., Mustala O. (1957). The effects of training on heart rate; a longitudinal study. Ann. Med. Exp. Biol. Fenn..

[26] Boukhris O., Abdessalem R., Ammar A., Hsouna H., Trabelsi K., Engel F.A., Sperlich B., Hill D.W., Chtourou H. (2019). Nap Opportunity During the Daytime Affects Performance and Perceived Exertion in 5-m Shuttle Run Test. Front. Physiol..

[27] Şahan A.E. (2017). Oryantiring Eğitiminin Dikkat ve Bellek Üzerine Etkisinin İncelenmesi. Ank. Üniversitesi Beden Eğitimi Spor Yüksekokulu SPORMETRE Beden Eğitimi Spor Bilim. Derg..

[28] Schaun G.Z., Ribeiro Y.S., Vaz M.S., Del Vecchio F.B. (2013). Correlation between Agility, Lower Limb Power and Performance in a Sport-Specific Test in Female Volleyball Players. Int. J. Sports Sci..

[29] Balsalobre-Fernández C. (2024). Real Time Estimation of Vertical Jump Height with a Markerless Motion Capture Smartphone App: A Proof-of-Concept Case Study. Proc. Inst. Mech. Eng. Part P J. Sports Eng. Technol..

[30] Cohen J. (1988). Statistical Power Analysis for the Behavioral Sciences.

[31] Ajjimaporn A., Ramyarangsi P., Siripornpanich V. (2020). Effects of a 20-Min Nap after Sleep Deprivation on Brain Activity and Soccer Performance. Int. J. Sports Med..

[32] Chtourou H., H’mida C., Boukhris O., Trabelsi K., Ammar A., Souissi N. (2019). Nap Opportunity as a Strategy to Improve Short-Term Repetitive Maximal Performance During the 5-m Shuttle Run Test: A Brief Review. Int. J. Sport Stu. Health.

[33] Dutheil F., Danini B., Bagheri R., Fantini M.L., Pereira B., Moustafa F., Trousselard M., Navel V. (2021). Effects of a Short Daytime Nap on the Cognitive Performance: A Systematic Review and Meta-Analysis. Int. J. Environ. Res. Public Health.

[34] Hayashi M., Watanabe M., Hori T. (1999). The Effects of a 20 Min Nap in the Mid-Afternoon on Mood, Performance and EEG Activity. Clin. Neurophysiol..

[35] Hsouna H., Boukhris O., Abdessalem R., Trabelsi K., Ammar A., Shephard R.J., Chtourou H. (2019). Effect of Different Nap Opportunity Durations on Short-Term Maximal Performance, Attention, Feelings, Muscle Soreness, Fatigue, Stress and Sleep. Physiol. Behav..

[36] Hsouna H., Boukhris O., Hill D.W., Abdessalem R., Trabelsi K., Ammar A., Irandoust K., Souissi N., Taheri M., Hammouda O. (2022). A Daytime 40-Min Nap Opportunity after a Simulated Late Evening Soccer Match Reduces the Perception of Fatigue and Improves 5-m Shuttle Run Performance. Res. Sports Med..

[37] Kubo T., Takeyama H., Matsumoto S., Ebara T., Murata K., Tachi N., Itani T. (2007). Impact of Nap Length, Nap Timing and Sleep Quality on Sustaining Early Morning Performance. Ind. Health.

[38] Suppiah H.T., Low C.Y., Choong G., Chia M. (2019). Effects of a Short Daytime Nap on Shooting and Sprint Performance in High-Level Adolescent Athletes. Int. J. Sports Physiol. Perform..

[39] Sheppard J.M., Cronin J.B., Gabbett T.J., McGuigan M.R., Etxebarria N., Newton R.U. (2008). Relative Importance of Strength, Power, and Anthropometric Measures to Jump Performance of Elite Volleyball Players. J. Strength Cond. Res..

[40] Pereira A., Costa A.M., Santos P., Figueiredo T., João P.V. (2015). Training Strategy of Explosive Strength in Young Female Volleyball Players. Medicina.

[41] Souabni M., Hammouda O., Souabni M.J., Romdhani M., Driss T. (2023). 40-Min Nap Opportunity Attenuates Heart Rate and Perceived Exertion and Improves Physical Specific Abilities in Elite Basketball Players. Res. Sports Med..

[42] Boukhris O., Trabelsi K., Suppiah H., Ammar A., Clark C.C.T., Jahrami H., Chtourou H., Driller M. (2024). The Impact of Daytime Napping Following Normal Night-Time Sleep on Physical Performance: A Systematic Review, Meta-Analysis and Meta-Regression. Sports Med..

[43] Fang W., Le S., Han W., Peng-Jiao X., Shuai Y., Rui-Ling Z., Lin L., Ya-Hui X. (2023). Association between Napping and Cognitive Impairment: A Systematic Review and Meta-Analysis. Sleep Med..

[44] Boukhris O., Trabelsi K., Ammar A., Abdessalem R., Hsouna H., Glenn J.M., Bott N., Driss T., Souissi N., Hammouda O. (2020). A 90 Min Daytime Nap Opportunity Is Better Than 40 Min for Cognitive and Physical Performance. Int. J. Environ. Res. Public Health.

[45] Mierzejewska-Krzyżowska B., Drzymała-Celichowska H., Celichowski J. (2011). Gender Differences in the Morphometric Properties of Muscle Fibres and the Innervation Ratio of Motor Units in Rat Medial Gastrocnemius Muscle. Anat. Histol. Embryol..

[46] Landen S., Hiam D., Voisin S., Jacques M., Lamon S., Eynon N. (2023). Physiological and Molecular Sex Differences in Human Skeletal Muscle in Response to Exercise Training. J. Physiol..

[47] Maki P.M., Sundermann E. (2009). Hormone Therapy and Cognitive Function. Hum. Reprod. Update.

[48] Vermeersch H., T’Sjoen G., Kaufman J.M., Vincke J., Van Houtte M. (2010). Gender ideology, same-sex peer group affiliation and the relationship between testosterone and dominance in adolescent boys and girls. J. Biosoc. Sci..

[49] Downing K., Chan S., Downing W., Kwong T., Lam T. (2008). Measuring Gender Differences in Cognitive Functioning. Multicult. Educ. Technol. J..

[50] Halpern D.F. (2000). Sex Differences in Cognitive Abilities.

[51] Ruigrok A.N.V., Salimi-Khorshidi G., Lai M.-C., Baron-Cohen S., Lombardo M.V., Tait R.J., Suckling J. (2014). A Meta-Analysis of Sex Differences in Human Brain Structure. Neurosci. Biobehav. Rev..

[52] Kimura D. (2002). Sex hormones influence human cognitive pattern. Neuro. Endocrinol. Lett..

[53] Adanur O., Eken Ö. (2024). Enhancing Anaerobic Performance in Kickboxers: The Strategic Role of Short-Duration Napping. Spor. Bilim. Araştırmaları Derg..

